# Prediction of electro-anatomical substrate and arrhythmia recurrences using APPLE, DR-FLASH and MB-LATER scores in patients with atrial fibrillation undergoing catheter ablation

**DOI:** 10.1038/s41598-018-31133-x

**Published:** 2018-08-23

**Authors:** Jelena Kornej, Katja Schumacher, Borislav Dinov, Falco Kosich, Philipp Sommer, Arash Arya, Daniela Husser, Andreas Bollmann, Gregory Y. H. Lip, Gerhard Hindricks

**Affiliations:** 1 Department of Electrophysiology, Heart Center, Leipzig, Germany; 2 University of Leipzig, Institute for Medical Informatics, Statistics, and Epidemiology, Leipzig, Germany; 3 Institute of Cardiovascular Sciences, University of Birmingham, Birmingham, UK

## Abstract

Arrhythmia recurrences after catheter ablation of atrial fibrillation (AF) cause intensive treatment costs. Left atrial electro-anatomical remodeling measured as low voltage areas (LVA) during catheter ablation indicates advanced disease stage and is associated with poor ablation success. The aim of this study was to analyze the prediction of LVA and arrhythmia recurrences using APPLE, DR-FLASH and MB-LATER scores. APPLE, DR-FLASH scores were calculated at baseline and MB-LATER at 3 months post-ablation in AF patients undergoing first catheter ablation. LVA was determined using high-density maps and defined as <0.5 mV. Early (ERAF, <3 months) and late (LRAF, 3–12 months) were analyzed during follow-up. The study population included 241 patients (age 64 ± 11 years, 59% males, 59% persistent AF, 27% LVA, 27% LRAF). LVA were significantly associated with recurrences (OR 2.081, p = 0.026). While on univariable analysis, all scores were significantly associated with LVA, on multivariable analysis only APPLE (OR 1.789, p < 0.001) and DR-FLASH (OR 2.144, p < 0.001) remained significant predictors. However, MB-LATER (OR 1.445, p = 0.034) and ERAF (OR 5.078, p < 0.001) remained associated with LRAF on the multivariable analysis. These results were validated in a subgroup of 873 patients (age 61 ± 10, 63% males, 39% persistent AF, 34% LRAF, 27% LVA) from The Leipzig Heart Center AF Ablation Registry. All scores were significantly associated with recurrences. However, ERAF was the most powerful predictor for later rhythm outcomes. Summarizing, a clinical score useful for prediction for both LVA and rhythm outcomes in AF patients remains a clinical unmet need.

## Introduction

Atrial fibrillation (AF) is the most common cardiac arrhythmia, which is associated with an increased risk of dementia, heart failure and thromboembolism, leading to an increased mortality^1^. Pathophysiological, AF results in electrical and later structural remodeling of the atrial myocardium (inflammation, fibrosis, and atrial dilatation)^2^.

Left atrial low voltage areas (LVA), also known as electro-anatomical substrate, represent these atrial remodeling processes and are considered to play an important role in AF progression^3,4^. LVA can be found in 10% of patients with paroxysmal AF and in 35% of patients with persistent AF^4^. However, performing individually tailored substrate modification, a significantly higher arrhythmia-free survival rate compared with a conventional approach can be achieved^5^.

Nevertheless, the prediction of LVA using clinical scores is understudied. Ribo *et al*. analyzed prediction of electro-anatomical substrate using the CHA_2_DS_2_-VASc score and demonstrated that high scores were associated with extensive AF substrate and higher recurrence rates after catheter ablation of persistent AF^6^. The DR-FLASH score (based on **D**iabetes mellitus, **R**enal dysfunction, persistent A**F** type, **L**A diameter >45 mm, **A**ge >65 years, female **S**ex, and **H**ypertension) was recently introduced to predict LVA and demonstrated a high predictive ability (AUC = 0.801, p < 0.001)^7^. Recently, we introduced the APPLE score (one point for **A**ge >65 years, **P**ersistent AF, im**P**aired eGFR (<60 ml/min/1.73 m2), **L**A diameter ≥43 mm, **E**F <50%) for the prediction of AF recurrences after catheter ablation in patients undergoing first and re-do intervention^8,9^. Later, we demonstrated that the APPLE score was useful to predict LVA, too^10^. The MB-LATER score (one point for **M**ale gender, **B**undle branch block or QRS >120 ms, **L**A diameter ≥47 mm, **A**F **T**ype (persistent AF), **E**arly **R**ecurrence <3 months) had been developed as the rhythm outcomes score for the prediction of very late arrhythmia recurrences (>12 months) after catheter ablation^11^, but its potential to predict the presence of LVA during catheter ablation is unknown. Therefore, the aim of the current study was to compare prediction of LVA and arrhythmia recurrences using the APPLE, MB-LATER and DR-FLASH scores in AF patients undergoing first catheter ablation.

## Methods

The study population included 241 AF patients from the BioAF cohort initiated at the Heart Center Leipzig between 2015 and 2017 as previously described^12^. All patients underwent first AF radiofrequency catheter ablation and were detailed phenotyped including clinical, imaging, peri-interventional, follow-up data and biodata (blood and plasma). Furthermore, a subgroup of 873 patients from The Leipzig Heart Center AF Ablation Registry (2007–2011) had been used to validate the results^8,13^. Exclusion criteria in both cohorts were pregnancy, age <18 or >75, valvular AF, cancer, acute or systemic inflammatory diseases. The study was approved by the local Ethical Committee (Medical Faculty, Leipzig University) and all patients provided written informed consent for participation. All methods were performed in accordance with the relevant guidelines and regulations.

Paroxysmal and persistent AF were defined according to current guidelines^1^. Paroxysmal AF was defined as self-terminating within 7 days after onset. Persistent AF lasted longer than 7 days or required drugs or electrical cardioversion for termination. In all patients, transthoracic and transoesophageal echocardiography were performed prior to the ablation. All class I or III antiarrhythmic medications with exception of amiodarone were discontinued for at least 5 half-lives before the AF ablation procedure.

### Catheter ablation

The electro-anatomical mapping was performed in sinus rhythm. In patients presenting with AF, the arrhythmia was terminated by electrical cardioversion and the mapping was further performed in sinus rhythm. End-point of the catheter ablation was isolation of the pulmonary veins with proof of both exit and entrance block. The electro-anatomical voltage maps of the left atrium excluding the pulmonary veins were created using multielectrode spiral catheter with interelectrode distance 2-5-2 or ablation catheter with a 3.5 mm electrode tip and contact measurement properties (SmartTouch Thermocool, Biosense Webster, Diamond Bar, CA, USA and TactiCath, St Jude Medical (SJM), Saint Paul, MN, USA) as mapping catheter. Electro-anatomical mapping was performed using 3-D electro-anatomical mapping systems (Carto, Biosense Webster, Diamond Bar, CA, USA or EnSite Precision, SJM). In both mapping systems the cut-off values for defining LVA were identical: <0.5 mV for low voltage and <0.2 mV for dense scar. Patients underwent high density mapping of left atrial voltage using multipolar catheters in combination with auto-annotation algorithms (AutoMap in Precision and ConfiDense in Carto 3). Here the number of points was >1000. Points with insufficient catheter-to-tissue contact or inside ablation lines were excluded. At the end of the procedure, an attempt to induce AF or left atrial macro-reentry tachycardia (LAMRT) was performed using a standardized protocol (burst stimulation with 300, 250, 200 ms from coronary sinus). According to the underlying LVA and inducible LAMRT additional ablation lines were applied.

In the subgroup from The Leipzig Heart Center AF Ablation Registry, LA catheter ablation was performed using a well-documented approach^13^. Briefly, in all patients, circumferential LA ablation lines were placed around the antrum of the ipsilateral pulmonary veins (irrigated tip catheter, pre-selected tip temperature of 48 °C, and maximum power of 30–50 W). In patients with persistent AF, additional linear lesions were added at the LA roof, the basal posterior wall and the LA (mitral) isthmus. At the end of procedure, linear block was confirmed across the roof and the mitral isthmus. The isolation of all pulmonary veins with bidirectional block was verified with a multipolar circular mapping catheter and was defined as the procedural endpoint.

In both cohorts, after ablation class I and III antiarrhythmic drugs were not routinely initiated. Only patients with failed sinus rhythm restoration received previous antiarrhythmic medication during blanking period and thereafter dependent on the rhythm in Holter ECG during follow-up. Proton pump inhibitors were added for 4 weeks. According to the guidelines^1^, oral anticoagulation was prescribed for 3–6 months after catheter ablation depending on risk stratification of stroke using the CHA_2_DS_2_-VASc score thereafter.

### Follow-up

All patients were followed in the outpatient clinic after catheter ablation. During the follow-up period, 4-days (in the BioAF cohort) or 7-days (in The Leipzig Heart Center AF Ablation Registry) Holter ECG recordings were performed at 3, 6 and 12 months. Additional ECGs and Holter ECG recordings were obtained when patients’ symptoms were suggestive of AF. Early arrhythmia recurrences (ERAF) were defined as any atrial arrhythmia lasting longer than 30 seconds and occurring within the first 3 months after procedure. Late arrhythmia recurrences (LRAF) were any atrial arrhythmia lasting >30 s between 3 and 12 months after ablation. If electrical or pharmacologic cardioversion and/or repeat procedure were needed after 3 months blanking period, this was also considered as an arrhythmia recurrence, i.e. study endpoint.

### Statistical analysis

Data are presented as mean and standard deviation (SD) if normally distributed or as median [interquartile range] for skewed continuous variables and as proportions for categorical variables. Continuous variables were tested for normal distribution using the Kolmogorov-Smirnov test. The differences between continuous values were assessed using an unpaired t-test if normally distributed or Mann–Whitney test if skewed continuous variables, and a chi-square test for categorical variables.

Multivariable logistic regression analysis (MV), which included variables with a p-value < 0.1 found on univariable analysis, was performed to identify predictors for LVA and arrhythmia recurrences. We performed multivariable analyses separately for APPLE, MB-LATER and DR-FLASH scores with adjustment for the variables, which were significant in univariable model, but were not included into the scores: Model 1 for the APPLE with adjustment for gender, hypertension, diabetes, bundle brunch block, ERAF; Model 2 for the MB-LATER with adjustment for hypertension, diabetes, EF, eGFR; Model 3 for the DR-FLASH with adjustment for EF, bundle brunch block, ERAF. In patients with available LVA data, LVA was adjusted to each model analyzing prediction of arrhythmia recurrences.

ROC (receiver operating characteristic) curves were generated for graphical illustration of APPLE, MB-LATER and DR-FLASH scores’ performance in predicting LVA and arrhythmia recurrences, with the area under the curve (AUC) being equivalent to the c-index for determining the predictive value for a score.

A p-value < 0.05 was considered statistically significant. All analyses were performed with IBM SPSS Statistics for Windows Version 23 (IBM Corp, Armonk, NY, USA).

## Results

### Study cohorts

The study population consisted of two cohorts: 241 patients from BioAF cohort with available LVA and rhythm outcomes data and 873 patients from The Leipzig Heart Center AF Ablation Registry used for the results validation (215 with available LVA data). The baseline characteristics of both cohorts are presented in Tables 1 and 2. Patients in the BioAF cohort were significantly older, had more often persistent AF, worse renal function and left ventricular EF, higher BMI, larger LA diameter, hypertension as well as higher APPLE and DR-FLASH scores than patients from The Leipzig Heart Center AF Ablation Registry (all p < 0.05, Suppl. Table 1). Patients in The Leipzig Heart Center AF Ablation Registry had significantly higher incidence for ERAF and LRAF.

**Table 1:** Baseline characteristics of the BioAF cohort (n = 241).

	LVA		*p*-value	Recurrences		*p*-value
	Yes (n = 65)	No (n = 176)		Yes (n = 62)	No (n = 179)	
Age, years	69 (64–75)	63 (55–71)	<0.001	64 (56–69)	65 (58–73)	0.112
Females	55	35	0.005	40	38	0.763
Persistent AF	82	51	<0.001	68	54	0.044
Electro-anatomical substrate	—	—	—	36	21	0.024
eGFR, ml/min/1.73 m^2^	68 (57–82)	79 (68–93)	<0.001	79 (66–89)	76 (63–89)	0.390
BMI, kg/m^2^	30 (26–33)	29 (26–33)	0.255	31 (27–34)	28 (26–33)	0.025
LA diameter, mm	42 (45–49)	43 (39–48)	0.028	45 (39–48)	44 (40–48)	0.765
EF, %	60 (50–65)	58 (50–65)	0.785	60 (50–65)	59 (50–65)	0.694
CHA2DS2-VASc score	3 (3–4)	2 (1–4)	<0.001	3 (1–4)	3 (1–4)	0.996
APPLE score	3 (2–4)	2 (1–2)	<0.001	2 (1–3)	2 (1–3)	0.579
DR-FLASH score	5 (4–5)	3 (2–4)	<0.001	4 (3–5)	4 (2–5)	0.214
MB-LATER score	2 (1–3)	2 (1–2)	0.053	2 (1–3)	2 (1–3)	0.018
Recurrences	22 (34)	37 (21)	0.024	—	—	—

Data presented as mean (IQR) or %.

Abbreviations: LVA – low voltage areas; AF – atrial fibrillation; BMI – body mass index; eGFR – estimated glomerular filtration rate; LA – left atrial; EF – ejection fraction; CHA_2_DS_2_-VASc score – congestive heart failure, hypertension, age ≥75 y, diabetes, stroke/thromboembolism, vascular disease (s), age 65–74 y, females; APPLE score – Age > 65 years, Persistent AF, imPaired eGFR (<60 ml/min/1.73 m2), LA diameter ≥43 mm, EF <50%; DR-FLASH score – diabetes mellitus, renal dysfunction, persistent form of AF, LA diameter >45 mm, age >65 years, female sex, and hypertension; MB-LATER score – Male gender, Bundle branch block or QRS >120 ms, LA diameter ≥47 mm, AF Type (persistent AF), Early Recurrence <3 months.

**Table 2:** Baseline characteristics of the validation cohort from The Leipzig Heart Center AF Ablation Registry (n = 873).

	Recurrences		*p*-value	LVA*		*p*-value
	Yes (n = 300)	No (n = 573)		Yes (n = 58)	No (n = 157)	
Age, years	63 (55–70)	61 (54–68)	0.007	69 (61–72)	60 (53–67)	<0.001
Females	39	35	0.212	36	19	0.009
Persistent AF	54	31	<0.001	86	57	<0.001
eGFR, ml/min/1.73 m^2^	95 (76–116)	97 (81–119)	0.100	72 (62–86)	83 (71–94)	<0.001
BMI, kg/m^2^	28 (26–31)	28 (25–31)	0.325	29 (27–31)	28 (27–31)	0.123
LA diameter, mm	44 (40–48)	42 (38–46)	<0.001	47 (40–49)	42 (39–47)	0.013
EF, %	60 (54–65)	60 (55–65)	0.040	58 (50–62)	60 (54–64)	0.145
CHA2DS2-VASc score	2 (1–3)	2 (1–3)	0.004	3 (2–4)	1 (1–2)	<0.001
APPLE score	2 (1–3)	1 (1–2)	<0.001	3 (2–4)	2 (1–2)	<0.001
DR-FLASH score	3 (2–4)	3 (2–4)	<0.001	5 (4–5)	3 (2–4)	<0.001
MB-LATER score	2 (1–3)	2 (1–2)	<0.001	3 (2–3)	2 (1–3)	0.035
Recurrences, %	—	—		43	35	0.277

*Subgroup from the Leipzig Heart Center AF Ablation Registry with available LVA data (n = 215).

### Prediction of LVA

LVA were significantly associated with LRAF (OR 2.081, 95% CI 1.092–3.965, p = 0.026). On the univariable analysis (Suppl. Table 2), age, female sex, persistent AF, hypertension, diabetes, renal function, LA diameter and all scores were significantly associated with LVA. However, on the multivariable analysis, only APPLE (OR 1.789, 95% CI 1.316–2.432, p < 0.001) and DR-FLASH (OR 2.144, 95% CI 1.587–2.896, p < 0.001) scores remained significant predictors for LVA, while MB-LATER did not reach significance (Suppl. Table 2).

**Figure 1:**
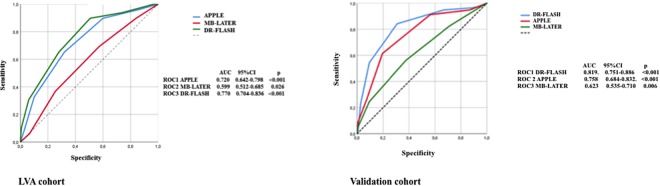
Prediction of LVA using APPLE, DR-FLASH and MB-LATER scores.

In the ROC curve analyses, all scores showed significant predictive value for LVA (APPLE AUC 0.720, 95% CI 0.642–0.798, p < 0.001, DR-FLASH AUC 0.770, 95% CI 0.704–0.836, p < 0.001, MB-LATER AUC 0.599, 95% CI 0.512–0.685, p = 0.026, Fig. 1).

In the validation cohort, all scores were significantly associated with LVA (Suppl. Table 3).

### Prediction of arrhythmia recurrences

#### BioAF cohort

There were 64 patients (27%) with LRAF in the BioAF cohort. On univariable analysis, persistent AF, LVA, ERAF and MB-LATER were significantly associated with rhythm outcomes within first 12 months after catheter ablation (Suppl. Table 4). On multivariable analyses, MB-LATER (OR 1.445, 95% CI 1.028–2.030, p = 0.034, MV Model 2) and ERAF (OR 5.078 in MV Model 1 and OR 5.066 in MV Model 3, both p < 0.001) remained significant predictors for LRAF.

#### The Leipzig Heart Center AF Ablation Registry

For the validation of predictive value of all scores we performed similar analyses in a subgroup of AF patients from The Heart Center Leipzig AF Ablation Registry. In total, 873 patients (age 61 ± 10, 63% males, 39% persistent AF, 34% LRAF) with available data for all scores’ calculation were included into analyses (Table 2).

On the univariable analysis (Suppl. Table 5), age, persistent AF, LA diameter, ERAF and all scores were significantly associated with LRAF. On the multivariable analysis, the APPLE (OR 1.550, 95% CI 1.333–1.803, p < 0.001), MB-LATER (OR 1.747, 95% CI 1.527–1.998, p < 0.001) and DR-FLASH (OR 1.242, 95% CI 1.118–1.381, p = 0.001) scores were significant predictors for LRAF. Similarly, as with LRAF prediction in the BioAF cohort, ERAF was the most powerful predictor for rhythm outcomes within 3–12 months with almost 4.5-fold risk for LRAF (OR 4.436 in MV Model 1 and OR 4.485 in MV Model 3, both p < 0.001).

**Figure 2:**
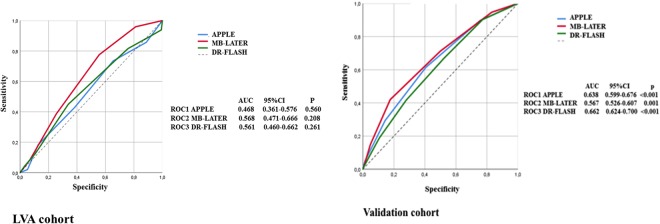
Prediction of recurrences using APPLE, DR-FLASH and MB-LATER scores.

In the ROC curve analysis, all scores demonstrated a significant predictive ability for LRAF: AUC 0.638, 95% CI 0.599–0.676, p < 0.001 for the APPLE score, AUC 0.662, 95% CI 0.624–0.700, p < 0.001 for DR-FLASH, and AUC 0.567, 95% CI 0.526–0.607, p = 0.001 for MB-LATER (Fig. 2).

## Discussion

To the best of our knowledge, this is the first analysis comparing the prediction for electro-anatomical remodelling measured as LVA and rhythm outcomes using APPLE, MB-LATER and DR-FLASH scores. First, the APPLE and DR-FLASH scores were significant predictors for LVA. Furthermore, all scores significantly predicted arrhythmia recurrences within 3–12 months after catheter ablation in a validation cohort from The Leipzig Heart Center AF Ablation Registry. However, the most powerful predictor for LRAF remained ERAF in both cohorts.

### Prediction of electro-anatomical substrate

Left atrial structural remodelling plays a major role in AF pathogenesis and up to date could be detected invasively using LA voltage mapping during catheter ablation^4,14^ or non-invasively using cardiac MRI^15^. Voltage-guided substrate modification by targeting LVA in addition to PVI is more effective than conventional PVI ablation approaches concerning arrhythmia freedom after the ablation^4,5,14^. Recently, Yagishita *et al*. showed that a LA voltage cut-off of <1.1 mV for electro-anatomic voltage mapping in sinus rhythm can be seen as an independent predictor for recurrences in patients without LVA (<0.5 mV)^16^. Although LVA is an important risk factor for post-procedural AF^4,5^, there are no standardized methods to predict LVA non-invasively before catheter ablation.

Some studies have analysed the prediction of electro-anatomical substrate using CHADS_2_ and CHA_2_DS_2_-VASc scores^6^, as both scores include the most important risk factors associated with cardiovascular complications in AF patients. Furthermore, the DR-FLASH score was developed to predict LVA in patients with AF undergoing radiofrequency catheter ablation^7^. However, clinical variables used by this score are mostly the same as in the widely used CHA_2_DS_2_-VASc score to predict thromboembolic risk. Nevertheless, it also includes variables as LA diameter and AF type, which are associated with AF progression.

The APPLE and MB-LATER scores were both introduced as rhythm outcomes predicting scores. While the APPLE score predicted arrhythmia recurrences within one year in patients undergoing first and repeat ablations^8,9^, the MB-LATER score had been developed for the prediction of arrhythmia recurrences over 12 months in patients with arrhythmia-free survival within first year^11^.

In the current study, we found that APPLE and DR-FLASH significantly predicted LVA before catheter ablation. The prediction of electro-anatomical substrate using DR-FLASH score was the best with more than 2-fold risk for LVA, however, rather expectable, as it is the score developed for the prediction of underlying electro-anatomical substrate in AF patients^7^. Of note, female gender – as a component of DR-FLASH score – was recently considered as a risk factor for substrate in AF patients. Indeed, females have a 2-fold risk for LVA^17^ and an almost 3-fold increased risk for AF recurrence following catheter ablation^3^. Females might probably present with clinical AF in a later state of fibro-fatty infiltration, which could explain a higher presence of electro-anatomical substrate and worse rhythm outcomes after catheter ablation^17^. These findings had been also confirmed in our current analysis.

In contrast, the APPLE had been developed as the rhythm outcomes prediction score and included such relevant components for AF progression as age, AF type and LA diameter^8,9^. Other components – like EF (an indicator for heart failure) and renal dysfunction – are also associated with an electro-anatomical substrate^18,19^. Therefore, the association between the APPLE score and underlying substrate in AF patients is explainable. This had been also recently confirmed^10^.

The MB-LATER score, however, did not reach significance predicting LVA. Although bundle branch block (BBB) – as the one of the parameters included in MB-LATER score – could be considered as a predictor for cardiac fibrotic turn-over in the ventricles mirroring remodelling processes in the atria, on multivariable analyses this parameter was not significantly associated with LVA. Interestingly, on multivariable analyses, hypertension was associated with almost 4-fold risk for LVA. Hypertension – as one of the most common cardiac diseases – leads to the cardiomyocyte hypertrophy and resulting pro-fibrotic changes^20,21^. Although in multivariable analyses it does not reach significance, importance of hypertension prevention and treatment in AF patients is obvious.

### Prediction of arrhythmia recurrences

Arrhythmia recurrences still remain a common issue after catheter ablation because of the impairment of the quality of life, higher hospitalization rates and consequently intensive treatment costs^1^. Therefore, on the one hand, there is a considerable clinical interest to predict the risk for recurrences already before procedure; on the other one – to shape personalized therapeutic strategies.

There have been multiple studies analysing the impact of different scores on recurrence prediction. First, the thromboembolic risk predicting CHADS_2_ and CHA_2_DS_2_-VASc scores demonstrated only modest prediction for arrhythmia recurrences^21,22^. This had led to the development of specific rhythm outcomes prediction scores as ALARMEC, BASE-AF2, CAAP-AF and ATLAS^23–26^. However, the results of these studies are partly difficult to interpret because of some non-standardized definitions (especially renal dysfunction and metabolic syndrome) and relatively small study populations. The APPLE score had been established for recurrences within 12 months after first ablation using 1.145 patients of The Leipzig Heart Center AF Ablation Registry^8^ as well as after repeat catheter ablation in 379 patients^9^. In both groups, APPLE score showed better ability to predict rhythm outcomes than CHADS_2_ and CHA_2_DS_2_-VASc. The MB-LATER score had been introduced to predict very late arrhythmia recurrences in patients with arrhythmia-free survival within 3–12 months after ablation^11^. It was developed using small retrospective cohort (n = 133), compared against other clinical scores (ALARMEc, BASE-AF_2_, APPLE, CHADS_2_, CHA_2_DS_2_–VASc, or HATCH), and demonstrated significantly better VLRAF prediction than most scores, apart from the APPLE score. Beside its predictive value for LVA, DR-FLASH was also associated with AF recurrences within 12 months after catheter ablation^7^, however in the initial study only cut-off had been shown as predictive.

In the current study, the ERAF demonstrated the best association with LRAF in the BioAF cohort and in the retrospective validation subgroup from The Leipzig Heart Center AF Ablation Registry. Of note, among all analysed scores, only MB-LATER included early recurrences^11^. Previously, we demonstrated that patients with ERAF had almost 4-fold risk for LRAF occurrence during follow-up^13^. This had been also confirmed by a meta-analysis^27^. However, ERAF is not available at baseline, therefore, MB-LATER cannot be calculated before catheter ablation.

### Prediction of electro-anatomical substrate and arrhythmia recurrences

In the current study, we found that the APPLE score demonstrated robust predictive value for both LVA and rhythm outcomes. On the one hand, it is a simple calculated score with easy obtainable clinical variables; on the other hand, it can be calculated already at baseline. Nevertheless, it was not significant for the prediction of arrhythmia recurrences in the BioAF cohort. Although both cohorts had been initiated in the same high-volume ablation center and performed using radiofrequency ablation approach, the BioAF cohort was initiated almost a decade later. This partly explains significant differences in baseline characteristics between both cohorts. While 10 years ago the guidelines recommended AF ablation only by failed rhythm and/or frequency control, during the last decade we got hard data presenting catheter ablation benefits on morbidity and mortality, what had led to the update of currently available AF management guidelines^1^. Also, during the last years catheter ablation had been more frequently recommended in older and comorbid patients. These observations explain why patients in the BioAF cohort were significantly older and had more co-morbidities. Also, there were significant differences in ERAF and LRAF occurrence between both cohorts. On the one hand, this could be explained by the technical development and application of better catheters and mapping systems in the BioAF cohort, on the other hand – by the shorter duration of ECG Holter monitoring during follow-up. Both issues might be the reason, why we failed to demonstrated significant prediction of arrhythmia outcomes using the APPLE score in BioAF cohort.

In general, prediction of arrhythmia recurrences remains complex as not only disease stage and patients’ comorbidities play a role, but also operators experience and continuity of the rhythm control. Still, prediction of electro-anatomical remodelling and rhythm outcomes remains challenging and obvious clinical unmet need. As both are very likely associated with pro-fibrotic changes in atrial myocardium, the prediction of atrial fibrosis using non-invasive tools (MRI, blood biomarkers) could influence therapy decisions and help to tailor individualized treatment strategy. Further translational and interdisciplinary studies are needed to elucidate the true role of atrial fibrosis and its’ prediction in AF patients.

### Study limitations

This study is limited by its observational, retrospective design in a small single-center cohort and retrospective results validation. Furthermore, the majority of the patients were not monitored by loop recorders; asymptomatic episodes of silent AF could have been missed. Also, there were only patients undergoing first radiofrequency AF catheter ablation. The results could differ in patients with repeated procedures or with ablation using other sources. Finally, in The Leipzig Heart Center AF Ablation Registry conducted between 2007 and 2011, the LVA data were available only in 214 patients.

## Conclusion

APPLE and DR-FLASH scores were significant predictors for LVA, while the most powerful predictor for the rhythm outcomes was ERAF. A clinical score useful for prediction of both LVA and rhythm outcomes after catheter ablation remains a clinical unmet need.

## Electronic supplementary material

Comparison of baseline characteristics between BioAF cohort and the validation cohort from The Leipzig Heart Center AF Ablation Registry

Prediction of LVA in the BioAF cohort

Prediction of LVA in the Leipzig Heart Center AF Ablation Registry

Prediction of arrhythmia recurrences in the BioAF cohort

Prediction of arrhythmia recurrences in the subgroup from the Leipzig Heart Center AF Ablation Registry

## Data Availability

All data generated or analyzed during this study are included in this published article (and its Supplementary Information files).
