# Supplementary material for: Prediction of electro-anatomical substrate and arrhythmia recurrences using APPLE, DR-FLASH and MB-LATER scores in patients with atrial fibrillation undergoing catheter ablation
Source: Sci Rep. 2018 Aug 23;8:12686. doi: 10.1038/s41598-018-31133-x (PMC6107514; doi:10.1038/s41598-018-31133-x)
Supplement: Supplementary file 1 — Comparison of baseline characteristics between BioAF cohort and the validation cohort from The Leipzig Heart Center AF Ablation Registry [file 41598_2018_31133_MOESM1_ESM.doc]

**Prediction of electro-anatomical substrate and arrhythmia recurrences using APPLE, DR-FLASH and MB-LATER scores in patients with atrial fibrillation undergoing catheter ablation**

Jelena Kornej,^1,2^ MD, MSc, Katja Schumacher,^1,3^ Borislav Dinov, MD,^1^ Falco Kosich,^1^ Philipp Sommer,^1^ MD, Arash Arya,^1^ MD, Daniela Husser,^1^ MD, Andreas Bollmann,^1^ MD, PhD, Gregory YH Lip,^3^ MD, Gerhard Hindricks,^1^ MD.

**Supplemental Table 1. Comparison of baseline characteristics between BioAF cohort and the validation cohort from The Leipzig Heart Center AF Ablation Registry**

|  | **BioAF cohort** | **Validation cohort** | ***p*-value** |
| --- | --- | --- | --- |
| **Age, years** | 65 (57-72) | 61 (54-68) | <0.001 |
| **Females, %** | 41 | 36 | 0.204 |
| **Persistent AF, %** | 59 | 39 | <0.001 |
| **eGFR, ml/min/1.73m2** | 77 (64-89) | 96 (79-118) | <0.001 |
| **BMI, kg/m²** | 29 (26-33) | 28 (25-31) | 0.018 |
| **LA diameter, mm** | 44 (40-48) | 42 (39-46) | 0.010 |
| **EF, %** | 56 (48-61) | 60 (55-65) | <0.001 |
| **BBB, %** | 8 | 7 | 0.652 |
| **ERAF, %** | 22 | 46 | <0.001 |
| **LRAF, %** | 27 | 34 | 0.028 |
| **Hypertension, %** | 81 | 74 | 0.043 |
| **Diabetes mellitus, %** | 22 | 18 | 0.100 |
| **CHADS-VASc score** | 2 (1-4) | 2 (1-3) | <0.001 |
| **APPLE score** | 2 (1-3) | 1 (1-2) | <0.001 |
| **DR-FLASH score** | 4 (3-5) | 3 (2-4) | <0.001 |
| **MB-LATER score** | 2 (1-3) | 2 (1-3) | 0.663 |

**Data presented as mean (IQR) or %**

**Abbreviations**: LVA – Low voltage areas; AF – atrial fibrillation; BMI – body mass index; eGFR – estimated glomerular filtration rate; LA – left atrial; EF – ejection fraction; CHA_2_DS_2_-VASc score – congestive heart failure, hypertension, age≥75y, diabetes, stroke/thromboembolism, vascular disease(s), age 65-74y, females; APPLE score – Age >65 years, Persistent AF, imPaired eGFR (<60 ml/min/1.73m2), LA diameter ≥43 mm, EF <50%; DR-FLASH score – diabetes mellitus, renal dysfunction, persistent form of AF, LA diameter >45 mm, age >65 years, female sex, and hypertension; MB-LATER score – Male gender, Bundle branch block or QRS >120ms, LA diameter ≥47mm, AF Type (persistent AF), Early Recurrence <3 months
