# Supplementary material for: Prediction of electro-anatomical substrate and arrhythmia recurrences using APPLE, DR-FLASH and MB-LATER scores in patients with atrial fibrillation undergoing catheter ablation
Source: Sci Rep. 2018 Aug 23;8:12686. doi: 10.1038/s41598-018-31133-x (PMC6107514; doi:10.1038/s41598-018-31133-x)
Supplement: Supplementary file 3 — Prediction of LVA in the Leipzig Heart Center AF Ablation Registry [file 41598_2018_31133_MOESM3_ESM.doc]

**Prediction of electro-anatomical substrate and arrhythmia recurrences using APPLE, DR-FLASH and MB-LATER scores in patients with atrial fibrillation undergoing catheter ablation**

Jelena Kornej,^1,2^ MD, MSc, Katja Schumacher,^1,3^ Borislav Dinov, MD,^1^ Falco Kosich,^1^ Philipp Sommer,^1^ MD, Arash Arya,^1^ MD, Daniela Husser,^1^ MD, Andreas Bollmann,^1^ MD, PhD, Gregory YH Lip,^3^ MD, Gerhard Hindricks,^1^ MD.

**Supplement Table 3. Prediction of LVA in the Leipzig Heart Center AF Ablation Registry (n=215)**

| **Variables** | **UV** | | | **MV Model 1** | | | **MV Model 2** | | | **MV Model 3** | | |
| --- | --- | --- | --- | --- | --- | --- | --- | --- | --- | --- | --- | --- |
|  | **OR** | **95%CI** | **p-value** | **OR** | 95%CI | p-value | OR | 95%CI | p-value | OR | 95%CI | p-value |
| **Age, y** | **1.102** | **1.059-1.146** | **<0.001** |  |  |  | 1.077 | 1.030-1.127 | 0.001 |  |  |  |
| **Females** | **2.403** | **1.233-4.682** | **0.010** | **3.020** | 1.371-6.650 | 0.006 |  |  |  |  |  |  |
| **Persistent AF** | **4.775** | **2.124-10.738** | **<0.001** |  |  |  |  |  |  |  |  |  |
| **Hypertension** | **5.424** | **1.855-15.860** | **0.002** | **4.134** | 1.266-13.500 | 0.019 | 3.214 | 1.025-10.073 | 0.045 |  |  |  |
| **Diabetes mellitus** | **3.074** | **1.405-6.725** | **0.005** | **1.641** | 0.647-4.161 | 0.297 | 1.922 | 0.774-4.776 | 0.159 |  |  |  |
| **eGFR, ml/min/1.73m²** | **0.967** | **0.950-0.985** | **<0.001** |  |  |  | 0.984 | 0.964-1.005 | 0.138 |  |  |  |
| **LA diameter, mm** | **1.061** | **1.010-1.115** | **0.018** |  |  |  |  |  |  |  |  |  |
| **EF, %** | **0.978** | **0.951-1.006** | **0.121** |  |  |  | 0.997 | 0.963-1.033 | 0.883 | 1.003 | 0.970-1.037 | 0.866 |
| **BBB** | **4.843** | **1.119-20.957** | **0.035** | **5.019** | 0.654-38.522 | 0.121 |  |  |  | 7.249 | 1.048-50.140 | 0.045 |
| **ERAF** | **1.305** | **0.713-2.389** | **0.387** | **1.215** | 0.595-2.480 | 0.592 |  |  |  | 1.183 | 0.572-2.446 | 0.651 |
| **APPLE** | **2.310** | **1.699-3.143** | **<0.001** | **2.347** | 1.680-3.281 | <0.001 |  |  |  |  |  |  |
| **MB-LATER** | **1.520** | **1.137-2.033** | **0.005** |  |  |  | 1.591 | 1.137-2.227 | 0.007 |  |  |  |
| **DR-FLASH** | **2.741** | **2.000-3.757** | **<0.001** |  |  |  |  |  |  | 2.712 | 1.954-3.765 | <0.001 |
