# Supplementary material for: Prediction of electro-anatomical substrate and arrhythmia recurrences using APPLE, DR-FLASH and MB-LATER scores in patients with atrial fibrillation undergoing catheter ablation
Source: Sci Rep. 2018 Aug 23;8:12686. doi: 10.1038/s41598-018-31133-x (PMC6107514; doi:10.1038/s41598-018-31133-x)
Supplement: Supplementary file 4 — Prediction of arrhythmia recurrences in the BioAF cohort [file 41598_2018_31133_MOESM4_ESM.doc]

**Prediction of electro-anatomical substrate and arrhythmia recurrences using APPLE, DR-FLASH and MB-LATER scores in patients with atrial fibrillation undergoing catheter ablation**

Jelena Kornej,^1,2^ MD, MSc, Katja Schumacher,^1,3^ Borislav Dinov, MD,^1^ Falco Kosich,^1^ Philipp Sommer,^1^ MD, Arash Arya,^1^ MD, Daniela Husser,^1^ MD, Andreas Bollmann,^1^ MD, PhD, Gregory YH Lip,^3^ MD, Gerhard Hindricks,^1^ MD.

**Supplement Table 4. Prediction of arrhythmia recurrences in the BioAF cohort (n=241)**

| **Variables** | **UV** | | | **MV Model 1** | | | **MV Model 2** | | | **MV Model 3** | | |
| --- | --- | --- | --- | --- | --- | --- | --- | --- | --- | --- | --- | --- |
|  | **OR** | **95%CI** | **p-value** | **OR** | **95%CI** | **p-value** | **OR** | **95%CI** | **p-value** | **OR** | **95%CI** | **p-value** |
| **Age, y** | 0.977 | 0.950-1.004 | 0.096 |  |  |  | 0.961 | 0.921-1.002 | 0.064 |  |  |  |
| **Females** | 0.911 | 0.498-1.668 | 0.763 | 0.660 | 0.308-1.415 | 0.285 |  |  |  |  |  |  |
| **Persistent AF** | 1.928 | 1.013-3.668 | 0.046 |  |  |  |  |  |  |  |  |  |
| **LVA** | 2.081 | 1.092-3.965 | 0.026 | 1.676 | 0.704-3.990 | 0.243 | 1.995 | 0.871-4.568 | 0.102 | 1.661 | 0.696-3.964 | 0.253 |
| **Hypertension** | 2.339 | 0.986-5.550 | 0.054 | 1.599 | 0.554-4.612 | 0.385 | 1.722 | 0.614-4.829 | 0.301 |  |  |  |
| **Diabetes mellitus** | 1.764 | 0.903-3.447 | 0.097 | 0.950 | 0.388-2.325 | 0.911 | 1.035 | 0.439-2.438 | 0.937 |  |  |  |
| **eGFR ml/min/1.73m²** | 1.002 | 0.986-1.018 | 0.841 |  |  |  | 1.000 | 0.976-1.023 | 0.972 |  |  |  |
| **LA diameter, mm** | 1.008 | 0.962-1.057 | 0.739 |  |  |  |  |  |  |  |  |  |
| **EF, %** | 0.986 | 0.957-1.017 | 0.384 |  |  |  | 1.004 | 0.968-1.042 | 0.813 | 0.983 | 0.948-1.018 | 0.334 |
| **BBB** | 0.883 | 0.273-2.856 | 0.835 | 0.689 | 0.122-3.873 | 0.672 |  |  |  | 0.787 | 0.145-4.264 | 0.781 |
| **ERAF** | 5.192 | 2.711-9.946 | <0.001 | 5.189 | 2.465-10.925 | <0.001 |  |  |  | 5.066 | 2.417-10.619 | <0.001 |
| **APPLE** | 1.063 | 0.831-1.358 | 0.627 | 0.967 | 0.706-1.326 | 0.837 |  |  |  |  |  |  |
| **MB-LATER** | 1.373 | 1.040-1.811 | 0.025 |  |  |  | 1.445 | 1.028-2.030 | 0.034 |  |  |  |
| **DR-FLASH** | 1.151 | 0.944-1.403 | 0.165 |  |  |  |  |  |  | 0.959 | 0.751-1.226 | 0.740 |

Univariable analysis presents unadjusted variables associated with arrhythmia recurrences in the BioAF cohort. Multivariable analyses had been performed separately for all scores with adjustment for the variables significant in univariable model, which were not included into the scores: **Model 1** for the APPLE with adjustment for LVA, gender, hypertension, diabetes mellitus, bundle brunch block and ERAF; **Model 2** for the MB-LATER with adjustment for LVA, hypertension, diabetes mellitus, EF and eGFR; **Model 3** for the DR-FLASH with adjustment for LVA, EF, bundle brunch block and ERAF.
