# Supplementary material for: Prediction of electro-anatomical substrate and arrhythmia recurrences using APPLE, DR-FLASH and MB-LATER scores in patients with atrial fibrillation undergoing catheter ablation
Source: Sci Rep. 2018 Aug 23;8:12686. doi: 10.1038/s41598-018-31133-x (PMC6107514; doi:10.1038/s41598-018-31133-x)
Supplement: Supplementary file 5 — Prediction of arrhythmia recurrences in the subgroup from the Leipzig Heart Center AF Ablation Registry [file 41598_2018_31133_MOESM5_ESM.doc]

**Prediction of electro-anatomical substrate and arrhythmia recurrences using APPLE, DR-FLASH and MB-LATER scores in patients with atrial fibrillation undergoing catheter ablation**

Jelena Kornej,^1,2^ MD, MSc, Katja Schumacher,^1,3^ Borislav Dinov, MD,^1^ Falco Kosich,^1^ Philipp Sommer,^1^ MD, Arash Arya,^1^ MD, Daniela Husser,^1^ MD, Andreas Bollmann,^1^ MD, PhD, Gregory YH Lip,^3^ MD, Gerhard Hindricks,^1^ MD.

**Supplement Table 5. Prediction of arrhythmia recurrences in the subgroup from the Leipzig Heart Center AF Ablation Registry (n=873)**

| **Variables** | **UV** | | | **MV Model 1** | | | **MV Model 2** | | | **MV Model 3** | | |
| --- | --- | --- | --- | --- | --- | --- | --- | --- | --- | --- | --- | --- |
|  | **OR** | **95%CI** | **p-value** | **OR** | **95%CI** | **p-value** | **OR** | **95%CI** | **p-value** | **OR** | **95%CI** | **p-value** |
| **Age, y** | 1.021 | 1.006-1.036 | 0.005 |  |  |  | 1.028 | 1.008-1.048 | 0.005 |  |  |  |
| **Females** | 0.832 | 0.623-1.111 | 0.213 | 1.314 | 0.953-1.813 | 0.096 |  |  |  |  |  |  |
| **Persistent AF** | 2.570 | 1.928-3.427 | <0.001 |  |  |  |  |  |  |  |  |  |
| **Hypertension** | 0.999 | 0.726-1.376 | 0.997 | 0.694 | 0.482-0.998 | 0.049 | 0.796 | 0.560-1.132 | 0.204 |  |  |  |
| **Diabetes mellitus** | 1.362 | 0.953-1.947 | 0.090 | 1.172 | 0.786-1.749 | 0.435 | 1.190 | 0.807-1.755 | 0.379 |  |  |  |
| **eGFR ml/min/1.73m²** | 0.996 | 0.991-1.000 | 0.062 |  |  |  | 1.000 | 0.994-1.005 | 0.966 |  |  |  |
| **LA diameter, mm** | 1.054 | 1.030-1.078 | <0.001 |  |  |  |  |  |  |  |  |  |
| **EF, %** | 1.001 | 1.000-1.002 | 0.151 |  |  |  | 1.001 | 1.000-1.002 | 0.154 | 1.001 | 1.000-1.003 | 0.081 |
| **BBB** | 1.297 | 0.759-2.218 | 0.342 | 1.149 | 0.631-2.095 | 0.649 |  |  |  | 1.265 | 0.707-2.265 | 0.428 |
| **ERAF** | 4.778 | 3.532-6.465 | <0.001 | 4.417 | 3.237-6.028 | <0.001 |  |  |  | 4.485 | 3.300-6.095 | <0.001 |
| **APPLE** | 1.624 | 1.415-1.863 | <0.001 | 1.550 | 1.333-1.803 | <0.001 |  |  |  |  |  |  |
| **MB-LATER** | 1.713 | 1.502-1.955 | <0.001 |  |  |  | 1.747 | 1.527-1.998 | <0.001 |  |  |  |
| **DR-FLASH** | 1.310 | 1.186-1.447 | <0.001 |  |  |  |  |  |  | 1.242 | 1.118-1.381 | 0.001 |
